# Development of two different formats of heterogeneous fluorescence immunoassay for bioanalysis of afatinib by employing fluorescence plate reader and KinExA 3200 immunosensor

**DOI:** 10.1038/s41598-019-51288-5

**Published:** 2019-10-14

**Authors:** Ibrahim A. Darwish, Haitham AlRabiah, Mohammed A. Hamidaddin

**Affiliations:** 1 Department of Pharmaceutical Chemistry, College of Pharmacy, King Saud University, P.O. Box 2457, Riyadh, 11451 Saudi Arabia; 2 Department of Medicinal and Analytical Chemistry, Faculty of Pharmacy, Sana’a University, Sana’a, Yemen

**Keywords:** Biochemical assays, Non-small-cell lung cancer

## Abstract

Afatinib (AFT) is a potent and highly selective drug used to treat various solid tumors including non-small cell lung cancer (NSCLC). To ensure safe and effective treatment of cancer patients with AFT, its plasma concentrations should be monitored. Thus, sensitive immunoassays are required for measuring AFT concentrations in plasma samples. In this study, two different formats of heterogeneous fluorescent immunoassays were developed and validated for AFT bioanalysis. These assays were microwell-based fluorescence immunoassay (FIA) using fluorescence plate reader and kinetic exclusion assay (KinExA) using KinExA 3200 immunosensor. Both FIA and KinExA were developed using the same reagents: mouse anti-AFT antibody, solid-phase immobilized AFT conjugated with bovine serum albumin protein (AFT-BSA), and goat anti-mouse IgG labelled with fluorescein isothiocyanate (FITC-IgG) for signal generation. The analytical performances of both assays were comparatively evaluated, and the results revealed that although both assays had comparable accuracies, KinExA was superior to FIA in terms of sensitivity and precisions. Moreover, both FIA and KinExA were better alternatives to the existing chromatographic methods for bioanalysis of AFT. The proposed FIA and KinExA are anticipated to effectively contribute in ensuring safe and effective treatment with AFT in clinical settings.

## Introduction

Afatinib (AFT) is a potent and highly selective drug used for treating various of solid tumors, including non-small cell lung cancer (NSCLC). It belongs to the tyrosine kinase inhibitor (TKI) drugs of the ErbB receptors family. It irreversibly inhibits signaling from all ErbB family receptors with high selectivity^1,2^. These receptors are essential for the proliferation, differentiation, and apoptosis of tumor cells; thus, their inhibition by AFT prevents the growth and spread of tumor cells, including mutation-positive of epidermal growth factor receptor (EGFR−) NSCLC and metastatic head and neck cancers^3^. On July 2013, the United States Food and Drug Administration (US-FDA) has approved AFT, as its dimaleate salt form, as the first-line treatment of metastatic NSCLC^4^. This drug is manufactured by the pharmaceutical company Boehringer Ingelheim Pharmaceuticals, Inc. (Ridgefield, USA) and marketed under the brand name of Gilotrif tablets. Gilotrif tablets are available in 20, 30, and 40 mg of AFT (equivalent to 29.56, 44.34, and 59.12 mg AFT dimaleate, respectively). After oral administration of Gilotrif tablets, the maximum plasma concentration is achieved in 2–5 h. Its maximum plasma concentration is dose-dependent in the range of 20–50 mg of AFT. However, high-fat diet decreases the plasma concentration of AFT by ~50%. The steady-state concentration of AFT in plasma is attained within 8 days of the repeated dose. The mean relative bioavailability of AFT oral solution containing 20 mg AFT is not significantly better than that of the oral 20 mg-Gilotrif tablets whose mean relative bioavailability is 92% of the oral solution^5^.

AFT showed potent therapeutic effects in clinical settings; however, as other TKIs, it showed low therapeutic index (narrow range between the therapeutic and toxic concentrations). Moreover, patients treated with AFT showed wide variability in AFT plasma concentrations despite receiving the same doses of AFT. Accordingly, wide variation in exposures to AFT occurs in treated patients^6^. In addition, it has been documented that AFT may cause abortion at the late gestational stages during pregnancy unless its dose is adjusted based on its measured plasma concentration. Furthermore, patients suffering from renal or hepatic impairment receiving AFT should be carefully monitored and their doses should be adjusted according to their own tolerance to the drug^1,5^. For these reasons, plasma AFT concentrations should be determined in patients during therapy to achieve the highest treatment efficacy and safety, and to avoid any potential adverse effects^7,8^. In addition, measurement of plasma AFT concentrations during therapy can elucidate total treatment failure or decreased responses in patients treated with AFT^9^; the reported concentration of AFT in plasma was ~58.9 ng mL^−1^. Thus, a sensitive and accurate bioanalytical assay with high throughput is required to support measurement of plasma AFT concentrations.

AFT has been determined in human plasma mostly by liquid chromatography with tandem mass spectrometry (LC-MS/MS)^10,11^. LC-MS/MS is a potential tool in bioanalysis of drugs; however, it has some limitations such as occurrence of “isobaric” interferences and ion suppression effect which negatively affect the assay selectivity. In addition, the highly complexed instrumentation and cost limit its routine use in clinical laboratories^12^. Immunoassays are more powerful alternatives for bioanalysis of drugs because of their inherent high selectivity for the analytes, low cost, simple procedures for sample pretreatments and/or analysis, and ability to process large number of samples; thus, they are suitable for application in clinical laboratories^13–15^. For these reasons, we were interested in developing immunoassays for bioanalysis of AFT. A previous study^16^ in our laboratory has described the production of a polyclonal antibody recognizing AFT with high selectivity and its use in the developed an enzyme-linked immunosorbent assay (ELISA) for AFT. However, heterogeneous microwell-based fluorescence immunoassay (FIA) with fluorescence plate reader is more powerful than ELISA because it is characterized with of higher sensitivity, time-saving in samples processing, wide working assay range, and high precision^17–19^. Furthermore, kinetic exclusion assay (KinExA) developed using KinExA immunosensor offers the same advantages as those of FIA in addition to convenient automation, and devoid from the common problems and limitations of ELISA^20–22^. Therefore, the present study aimed to develop microwell-based FIA using a fluorescence plate reader and KinExA using a KinExA 3200 immunosensor for bioanalysis of AFT.

## Experimental

### Instruments

Microplate fluorescence reader (FLx800) and automatic microplate strip washer (ELx50) were products of Bio-Tek Instruments Inc. Winooski, USA). KinExA 3200 immunosensor was a product of Sapidyne Instruments Inc. (Boise, ID, USA). Incubator (MINI/18) was purchased from Genlab Ltd. (Widnes, UK). Milli-Q water purification system (Labo, Millipore Ltd., Bedford, USA). Double beam spectrophotometer (UV-1601 PC: Shimadzu, Kyoto, Japan) with matched 1-cm quartz cells. Nutating mixer (Taitec, Saitama-ken, Japan). Microprocessor laboratory pH meter (BT-500: Boeco, Hamburg, Germany).

### Materials

Afatinib (Shanghai Haoyuan Chemexpress Co., Ltd. Shanghai, China) was used as received; its purity was >99%. Mouse antibody recognizing AFT with high specificity was generated using quinazolinediamine derivative of AFT conjugated with keyhole limpet hemocyanin protein as an immunogen; details of its generation and characterization were described in a previous report by our laboratory^16^. Briefly, quinazolinediamine derivative of AFT was synthesized and subsequently coupled with keyhole limpet hemocyanin (KLH) protein by diazotization/coupling reaction. The immunogen (AFT-KLH) was used for immunization of BALB/c mice. The immunization protocol was approved by the Animal Ethics Committee of the Pharmacology Department, College of Pharmacy, King Saud University, Kingdom of Saudi Arabia (No. KSU-SE-17-17). All methods were performed in accordance with the King Saud University Guidelines for institutional animal care and use committee pertaining to the Institutional Animal Care and Use Committee (IACUC). After full immunization of the animal, crude serum was collected as an anti-AFT polyclonal antibody from the most appropriately responded to immunization. To ensure good conditions of the antibody, its binding characteristics (affinity and specificity) were assessed, and the results showed that the antibody characteristics were identical to those reported in the previous study^16^. 96-Wells white-opaque flat-bottom FIA plates were purchased from Corning/Costar Inc. (Corning, NY, USA). Fluorescein isothiocyanate conjugate of goat anti-mouse IgG (FITC-IgG) and bovine serum albumin (BSA) protein were obtained from Sigma-Aldrich LLC (St. Louis, CA, USA). Polymethyl methacrylate (PMMA) beads (140–170 mesh, 98 µm) were purchased from Sapidyne Instruments Inc. (Boise, ID, USA). Human plasma was obtained from blood bank at King Khalid University Hospital (Riyadh, Saudi Arabia) and was kept frozen at −20 °C until analysis. All other chemicals used in this study were of analytical grade.

### Procedures

#### Preparation and characterization of AFT-BSA conjugate

A BSA conjugate of AFT was prepared according to the procedures described by Al-Shehri *et al*.^16^. Briefly, quinazolinediamine derivative of AFT was converted to its diazonium salt, which was subsequently coupled with BSA protein via its tyrosine amino acid residues. The remaining molecules of the unconjugated quinazolinediamine derivative of AFT were removed from the AFT-BSA conjugate by dialysis. After dialysis, the conjugate was characterized by protein assay and ultraviolet (UV) spectrophotometry. The UV spectrum of the conjugate was compared with that of unconjugated BSA protein which was generated using the same concentrations under the same pH conditions. The shape and molar extinction coefficients of these spectra were examined to confirm successful formation of the conjugate.

#### Preparation of FITC-IgG solutions

Working solutions of goat anti-mouse FITC-IgG were prepared at concentrations of 0.4 and 0.25 µg mL^−1^ for use in FIA and KinExA, respectively. These solutions were prepared as described in our previous studies^19,20^. The solutions were prepared fresh at the start of each experiment.

#### Coating of AFT-BSA conjugate onto FIA plates and PMMA beads

For FIA plates, aliquots (50 µL) of AFT-BSA conjugate solution (2 μg mL^−1^) were dispensed into each well of the FIA plate. The plate was incubated for 2 h at 37 °C and then washed three times with PBS containing 0.05% Tween 20 by automatic microplate washer. The remaining protein binding sites on the plate wells were then blocked. For blocking, 100 µL of BSA solution (1%, w/v, in PBS) was dispensed into each well of the assay plate which was incubated for 30 min at 37 °C. Next, the plate was washed as described above, and then used to conduct FIA. The coated and blocked plates could be stored for at least 6 weeks at 4 or −20 °C without any noticeable deterioration of the coated AFT-BSA conjugate. The details of coating the FIA plates were described in our previous study^19^.

For PMMA beads, aliquots (1 mL) of AFT-BSA conjugate solution (1 µg mL^−1^) were added to PMMA beads. The beads suspension were gently agitated on a nutating mixer for 2 h at room temperature (25 ± 2 °C). The beads were allowed to settle down, and then the supernatant solution was decanted. The remaining binding sites on the beads were blocked by adding 1 mL of BSA solution (1%, w/v, in PBS) to the beads and the beads suspension was gently agitated for 1 h at room temperature. The coated and blocked beads were used to conduct KinExA or stored at 4 °C in blocking solution until used in analysis. The coated and blocked beads could be stored for one week without any noticeable deterioration. The details of coating the FIA plates were described in our previous study^20^.

#### Preparation of plasma samples for analysis by FIA and KinExA

Standard AFT was spiked in AFT-free plasma samples (50 μL) and the AFT-spiked samples were diluted to 2 mL with PBS or PBS containing BSA (1%, w/v) for analysis by FIA or KinExA, respectively. AFT was spiked to final concentration in the range of 0.1−200 and 0.01−100 ng mL^−1^ for FIA and KinExA, respectively^19,20^.

#### Procedures of FIA

The analysis by FIA was conducted according to the detailed procedures described in our previous study^19^. Briefly, aliquots (50 µL) of samples (standard solutions or spiked plasma samples) containing AFT (0.1 − 200 ng mL^−1^) were mixed with equal volumes of anti-AFT antibody (diluted 160-fold in PBS). An aliquot (50 µL) of the mixture was dispensed into each well of the assay plate that had been coated with AFT-BSA and blocked with BSA, as described above. The plate was kept in an incubator for 1 h at 37 °C, and then washed three times with PBS containing 0.05% Tween 20. Next, 50 µL of FITC-IgG solution (0.4 µg mL^−1^) was dispensed into each well of the plate which was subsequently incubated for 1 h at 37 °C. The plate was washed as before, and the fluorescence signals emitted from the bottom of the wells was directly measured by an FLx800™ microplate fluorescence reader at excitation ad emission filters of 485 and 528 nm, respectively.

#### Procedures of KinExA

Detailed features of the KinExA 3200 immunosensor and its setup for running the samples have been described in previous reports^20,21^. AF samples (0.01–100 ng mL^−1^) were mixed with equal volumes of anti-AFT antibody (diluted 160-fold in PBS). The mixture solutions were allowed to equilibrate for 30 min at room temperature and the samples (500 μL) were then allowed to pass through the PMMA beads coated with AFT-BSA by negative pressure of the instrument for 120 s at a rate of 0.25 mL min^−1^. Any excess materials were washed out by PBS. Next, 500 μL of anti-mouse FITC-IgG solution (2.5 µg mL^−1^) was drawn past the beads for 120 s at a rate of 0.25 mL min^−1^. Unbound FITC-IgG was washed out by flowing 1.5 mL of PBS through the bead-pack over a period of 90 s at a flow rate of 1 mL min^−1^. The bound FITC-IgG was counted and assessed by measuring fluorescence intensity. The data were acquired and processed as described in our previous report^20^.

## Results and Discussion

### Description of FIA and KinExA

**Figure 1:**
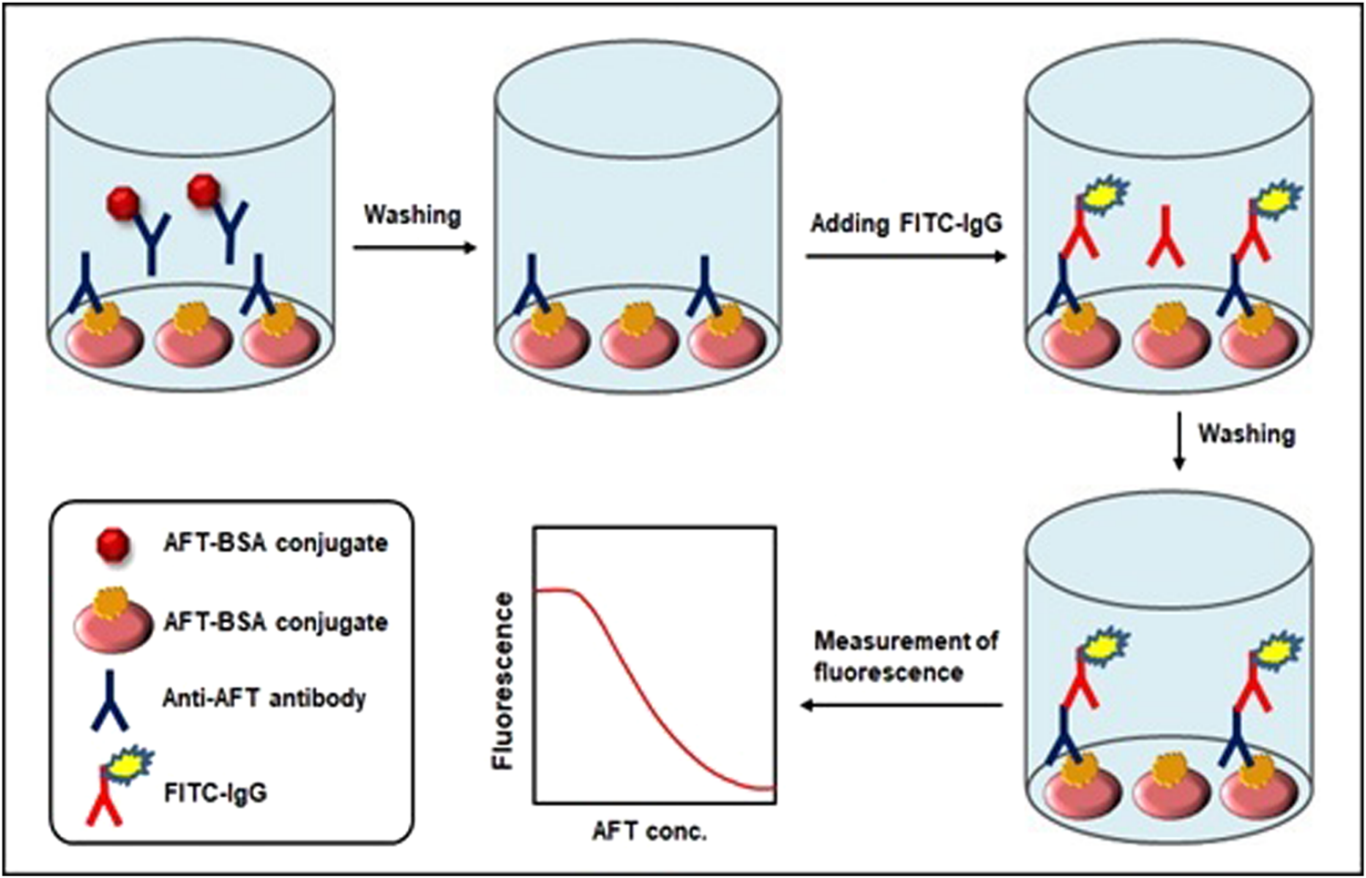
Schematic diagram for the proposed FIA for AFT.

**Figure 2:**
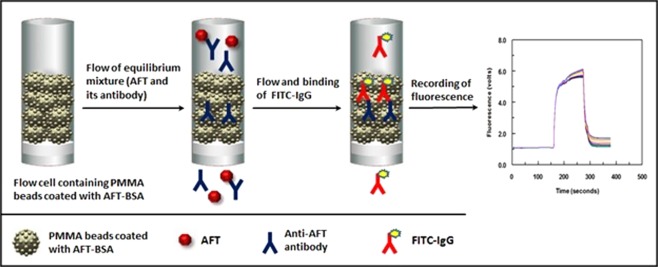
Schematic diagram for the proposed KinExA for AFT.

The features and technical procedures of the proposed FIA and KinExA are described in Figs 1 and 2, respectively. In FIA, free AFT (in sample) and immobilized AFT (AFT-BSA conjugate immobilized onto the FIA plate wells) were allowed to compete in binding to a predetermined quantity amount of antibody recognizing AFT. After completion of competitive binding reaction and removing the unbound materials by washing, the anti-AFT antibody bound to the plate wells was quantified by FITC-IgG reagent. The fluorescence signals emitted from the bottom of the wells were measured by a fluorescence reader. The measured fluorescence intensities were inversely proportional to the concentrations of AFT in the sample solutions.

In KinExA, a mixture of AFT and its specific antibody was allowed to pass rapidly over AFT-BSA coated on the PMMA beads. Free anti-AFT antibody molecules bound to the immobilized AFT-BSA; however, the complexes with AFT did not, and instead passed through the flow cell. During the flow of the mixture, the immune complex of AFT with its antibody did not dissociate; thus, free anti-AFT antibody molecules were kinetically excluded from binding to the immobilized AFT-BSA. The quantity of antibody bound to PMMA beads coated with AFT-BSA was then determined by FITC-IgG. Fluorescence signals were continuously monitored by the photodiode detector of the KinExA 3200 instrument, and a fluorescence-time plot (KinExAgram) was generated. The intensities of the fluorescence measured on the PMMA beads coated with AFT-BSA (the right-most plateau segment at the KinExAgram) were inversely correlated with AFT concentration in the original samples.

### Preparation and characterization of AFT-BSA conjugate

**Figure 3:**
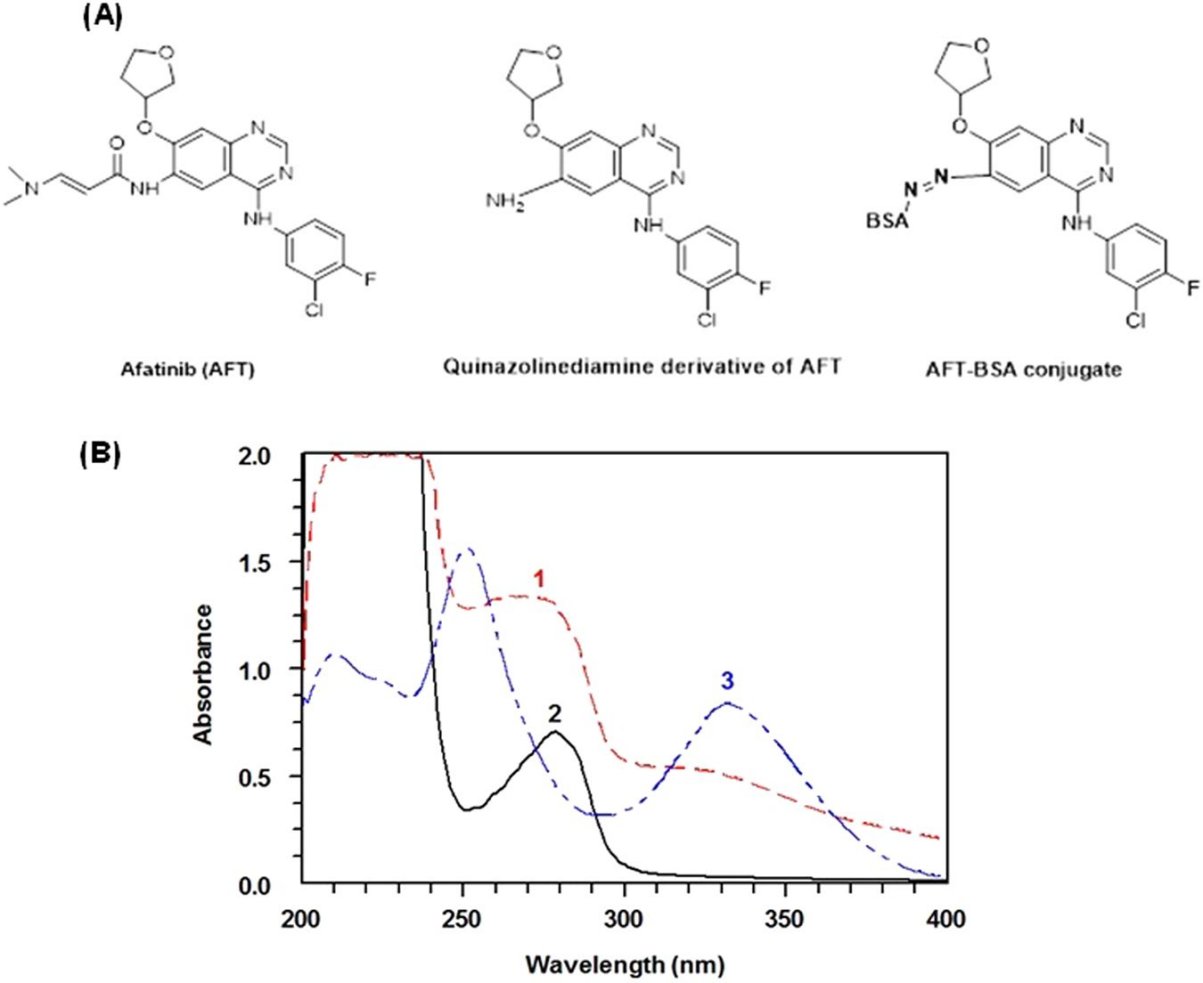
Panel (A) Chemical structures of AFT, AFT quinazolinediamine derivative and AFT-BSA conjugate. Panel (B) UV-absorption spectra for characterization of AFT-BSA conjugate. Spectra were: AFT quiazolinediamine derivative (1), BSA (2), and AFT-BSA conjugate (3). Concentrations were 0.1, 1.5, and 1.5 mg mL^−1^ for AFT quinazolinediamine, unconjugated BSA, and AFT-BSA conjugate, respectively.

AFT was converted to its quinazolinediamine derivative (Fig. 3A) by hydrolysis^16^. This derivative was isolated and subsequently conjugated with BSA to form AFT-BSA conjugate according to the procedures described in a previous report by our laboratory and^16^. To confirm the formation of AFT-BSA conjugate, UV spectral analysis was conducted. The absorption spectra of AFT-BSA and unconjugated BSA generated under the same conditions (Fig. 3B) were investigated. The absorption coefficient of AFT-BSA conjugate at its maximum absorption wavelength was significantly higher than that of unconjugated BSA protein. This hyperchromic effect in the conjugate spectrum was an evidence of the successful linking of the chromophoric molecules of quinazolinediamine derivative of AFT with BSA protein, as well as the formation of AFT-BSA conjugate. The coupling density of AFT-BSA conjugate was determined by spectrophotometric analysis according to the procedures described by Mastronicolis *et al*.^23^ assuming 6.6 × 10^4^ Daltons as the molecular weight of BSA. The average molecular ratio of AFT to BSA was determined to be 28.8

### Optimization of FIA and KinExA conditions

#### Selection of AFT-BSA concentration and anti-AFT antibody dilution

**Figure 4:**
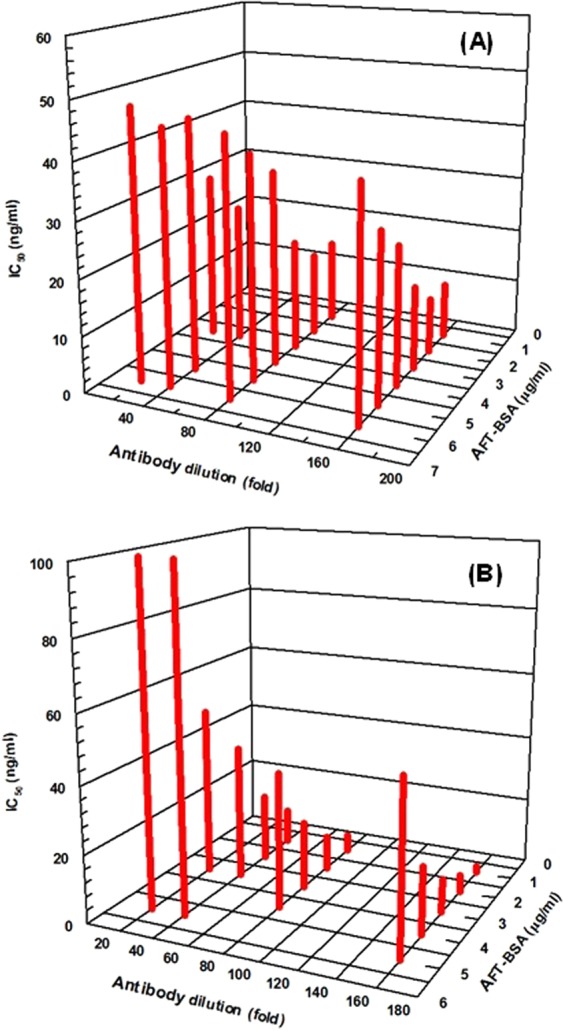
IC_50_ values obtained from the competitive FIA (**A**) and KinExA (**B**) of AFT at varying concentrations of AFT-BSA conjugate and varying dilutions of anti-AFT antibody.

Both FIA and KinExA were competitive assays; therefore, their highest sensitivities can be achieved when the amount of antibody is limited for a particular amount of immobilized AFT-BSA conjugate. To select the best combination of AFT-BSA concentration and anti-AFT antibody dilution, a series of competitive assays was carried out using varying concentrations of immobilized AFT-BSA and varying dilutions of anti-AFT antibody, with the IC_50_ value determined in each case (Fig. 4). In both FIA and KinExA, IC_50_ values decreased with increasing antibody dilution at a fixed AFT-BSA concentration. Similarly, IC_50_ decreased with deceasing AFT-BSA conjugate concentration at a fixed antibody dilution. The lowest IC_50_ values (the highest assay sensitivity) were achieved when the concentrations of AFT-BSA conjugate were 2 and 1 μg mL^−1^ for FIA and KinExA, respectively, and when the anti-AFT antibody dilution was 160-folds in both FIA and KinExA.

#### Immobilization of AFT-BSA onto microwells and PMMA beads and their blocking

**Figure 5:**
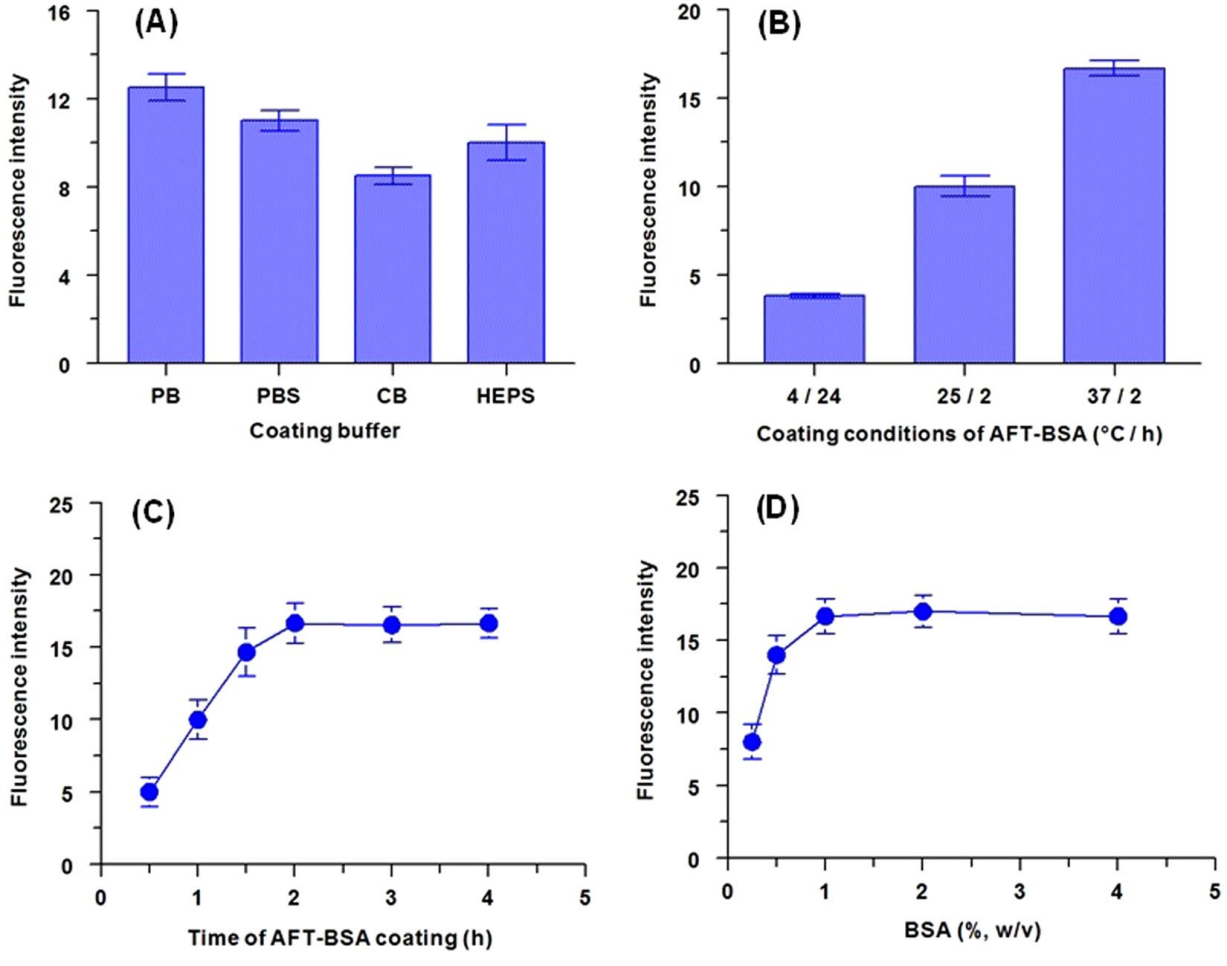
Effect of experimental conditions on the coating of AFT-BSA conjugate onto the microwells of the assay plate and their blocking with BSA protein. These conditions were: type of coating buffer (**A**), temperature and time for coating (**B**), time of coating at 37 °C (**C**), and concentration of BSA for blocking the microwells (**D**). Abbreviations are: PB: phosphate buffer, PBS: phosphate buffer saline, CB: carbonate buffer, and HEPS: 4-(2-hydroxyethyl)-1-piperazineethanesulfonic acid buffer. Values are the means of 3 determinations ± SD.

To select the best buffer for use in immobilizing AFT-BSA conjugate onto the FIA plate wells, four buffers were tested with varying pH values immobilization conditions (Fig. 5). These buffer solutions were phosphate buffer (PB), PBS, carbonate buffer (CB), and 4-(2-hydroxyethyl)-1-piperazine ethane sulfonic acid buffer (HEPS). The best immobilization was achieved by PB (Fig. 5A), the plates should be incubated for at least 2 h at 37 °C (Fig. 5B,C). To decrease non-specific binding and accordingly minimize background signals, the remaining binding sites on the FIA plate wells should be blocked with a blocking reagent. BSA was selected as a blocking agent because of its proven powerful blocking ability^24,25^. Our subsequent experiments proved that BSA solution (100 µL of 1%, w/v) of was sufficient for efficiently blocking the wells (Fig. 5D). Similar experiments were carried out for coating and blocking of PMMA beads for use in KinExA and the optimized conditions are summarized in Table 1.

**Table 1:** Summary for optimization of conditions for development of FIA and KinExA for AFT bioanalysis.

Parameter/condition	Optimum value	
	FIA	KinExA
Coating AFT-BSA conjugate conc. (μg mL^−1^)	2	1
Coating buffer type	PB	PBS
Coating time (h)/temperature (°C)	2/37	2/(25 ± 2)
BSA concentration for blocking (%, w/v)	1	1
Blocking with BSA: time (min)/temperature (°C)	30/37	1/(25 ± 2)
Binding of anti-AFT antibody: time (min)/temperature (°C)	60/37	30/(25 ± 2)
FITC-IgG conc. (μg mL^−1^)	0.4	2.5
Binding of FITC-IgG: time (h)/temperature (°C)	1/37	0.032/(25 ± 2)

#### Concentration of FITC-IgG and signal generation

**Figure 6:**
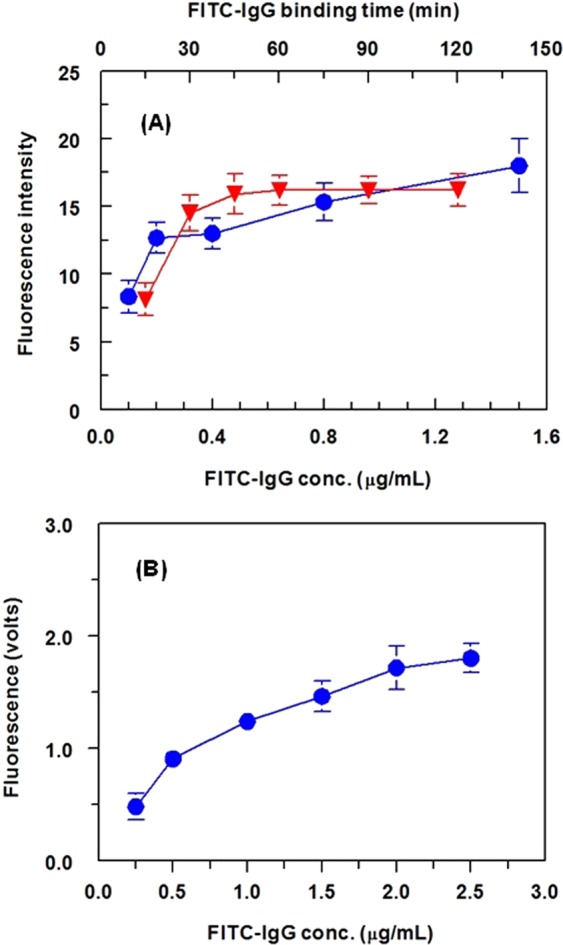
Panel (A) Effect of FITC-IgG concentration (●) and its binding time (▼) on the fluorescence signals generated from FIA. Panel (B): effect of FITC-IgG concentration on the fluorescence signals generated from KinExA. Values are the means of 3 determinations ± SD.

**Figure 7:**
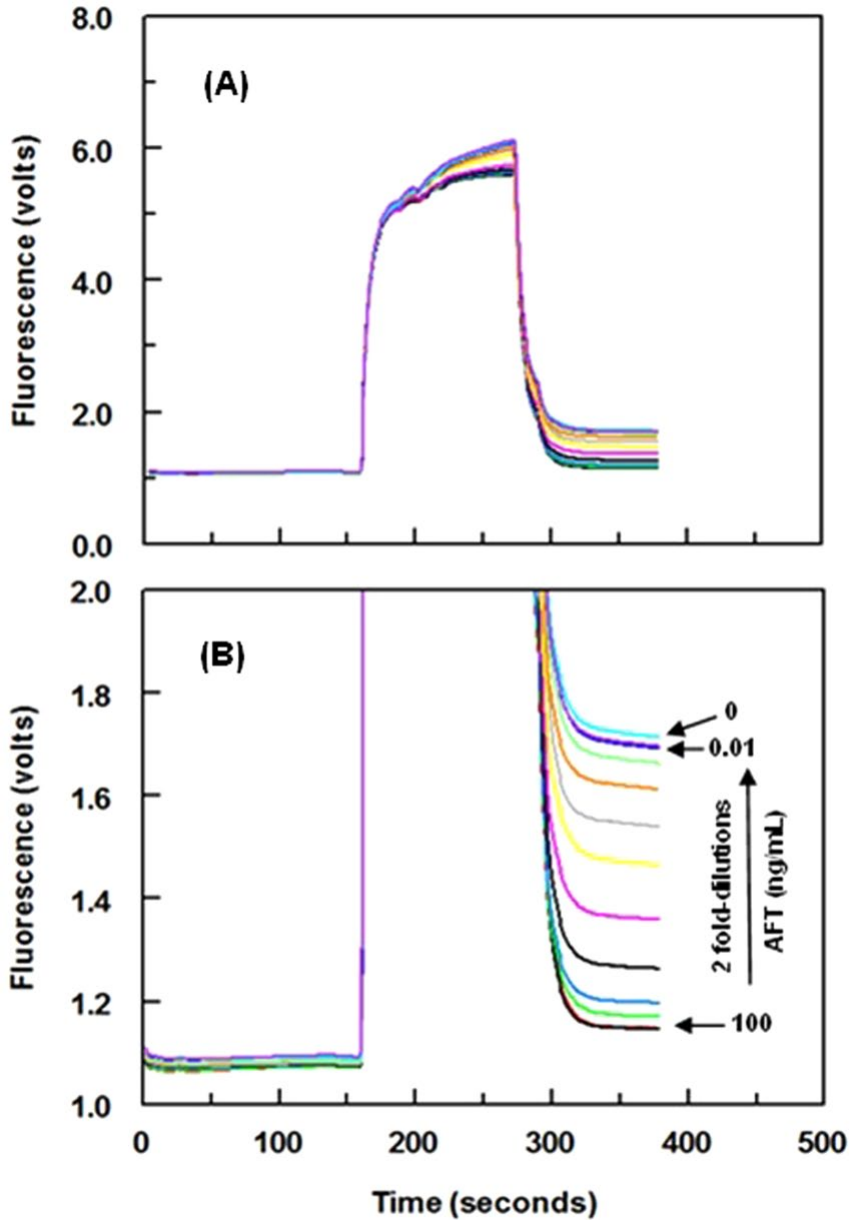
Panel (A) Real raw trend-line fluorescence responses (KinExAgram) obtained by the KinExA 3200 instrument for varying concentrations of AFT (0.01–100 ng mL^−1^). Panel (B) The same signals presented on different scale for the fluorescence (volts).

FITC-IgG has been widely used as a fluorescent label in different immunoassays because it provides highly sensitive assays. The best concentrations of FITC-IgG for generation of signals in both FIA and KinExA were established. For FIA, the optimum FITC-IgG concentration was 0.4 µg mL^−1^, with an optimum binding time of 1 h at 37 °C (Fig. 6A). For KinExA, the optimum FITC-IgG concentration was 2.5 µg mL^−1^ with a flow rate on the beads of 0.25 mL min^−1^. These conditions afforded the highest analytical fluorescence signals with the lowest background signals. Under optimum conditions of KinExA, the KinExAgram (instrument response versus time) for processing varying concentrations of AFT (0.01−100 ng mL^−1^) was generated (Fig. 7). The top-most curve in the KinExAgram (Fig. 7) corresponds to the blank solution (0 ng mL^−1^ AFT) and the bottom-most one corresponds to the highest AFT concentration (100 ng mL^−1^)

### Validation of FIA and KinExA

**Figure 8:**
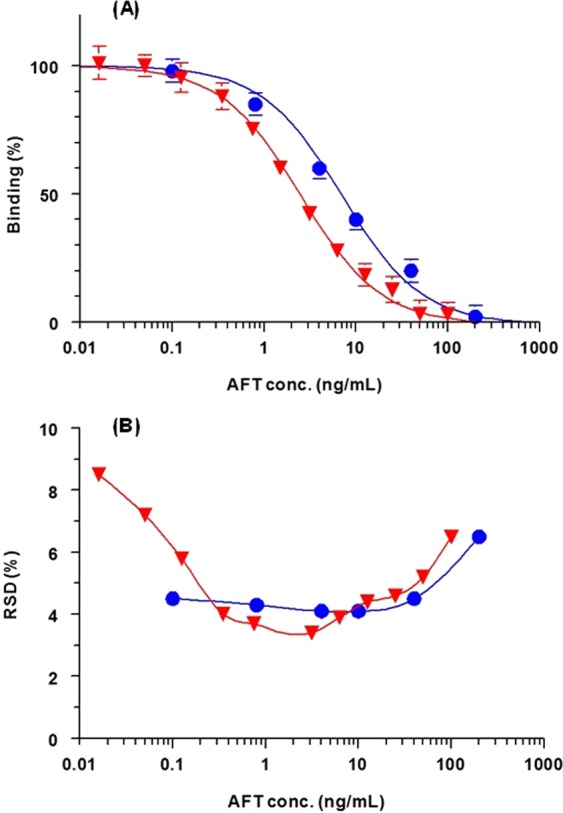
Calibration curves (**A**) and precision profiles (**B**) of the FIA (●) and KinExA (▼) for determination of AFT. RDS is the relative standard deviation.

Both FIA and KinExA were validated according to the validation guidelines of immunoassays for bioanalysis^26^. The calibration curves of both assays were generated using AFT at concentration ranges of 0.1−200 and 0.01−100 ng mL^−1^ for FIA and KinExA, respectively (Fig. 8A). The signals (binding %) correlated very well with AFT concentrations in the four-parameter curve fit; correlation coefficients (r) were 0.9989 and 0.9985 in FIA and KinExA, respectively. The limits of detection (LOD) and limits of quantitations (LOQ) were determined as described by Aanderson DJ^27^; LOD and LOQ = 3 σ and 10 σ, respectively [where σ is the standard deviation of the blank values (i.e. at 100% binding on the binding curve]. The LOD and LOQ of FIA were 0.4 and 1.2 ng mL^−1^, whereas those of KinExA were 0.1 and 0.3 ng mL^−1^. These high sensitivities were sufficient for AFT bioanalysis in plasma samples without preconcentration of samples prior to analysis because the reported plasma level of AFT was 58.9 ng mL^−1^ ^28^. The IC_10_ and IC_20_ values for FIA were 0.8 and 2 ng mL^−1^; however the values of IC_10_ and IC_20_ values for KinExA were 0.3 and 0.6 ng mL^−1^, respectively. The precision profiles of calibration concentrations of both assays are presented in Fig. 8B. Both FIA and KinExA afforded satisfactory intra- and inter-assay precisions at varying concentration levels (low, medium and high) of AFT with relative standard deviation (RSD) values of not exceeding 8.4% (Table 2). Recovery studies on AFT-spiked plasma samples revealed that the plasma matrix had negative effect on recovery values, and that the samples should be diluted at least 32-folds in PBS before analysis to afford acceptable recovery values. The high sensitivities of both assays allowed this dilution folds and enabled the use of very small volumes of plasma samples for the analysis. Recovery values from the diluted plasma samples were 99.1–103.5% and 95.5–103.4% in FIA and KinExA, respectively (Table 3). These values indicated the high accuracy of both FIA and KinExA for AFT bioanalysis.

**Table 2:** Precisions of the proposed FIA and KinExA for AFT at different concentration levels.

FIA			KinExA		
Concentration (ng mL^−1^)	Intra-assay	Inter-assay	Concentration (ng mL^−1^)	Intra-assay	Inter-assay
1	8.4	6.1	0.25	6.5	6.8
5	5.2	4.8	2.5	5.8	6.2
50	6.3	8.3	10	3.7	4.1

^a^Values are RSD (%); mean of 8 determinations.

**Table 3:** Analytical recovery of FIA and KinExA for bioanalysis of AFT spiked in human plasma samples.

FIA		KinExA	
Spiked AFT conc. (ng/mL)	Recovery (% ± RSD)^a^	Spiked AFT conc. (ng/mL)	Recovery (% ± RSD)^a^
2	103.5 ± 5.3	1.25	97.2 ± 5.6
5	99.1 ± 3.9	2.5	103.4 ± 4.2
10	102.3 ± 4.8	5	98.7 ± 4.1
20	103.1 ± 5.1	10	95.5 ± 4.2
40	100.9 ± 6.4	20	102.3 ± 5.7
	101.8 ± 1.8		99.4 ± 3.4

^a^Values are mean of 3 determinations.

**Figure 9:**
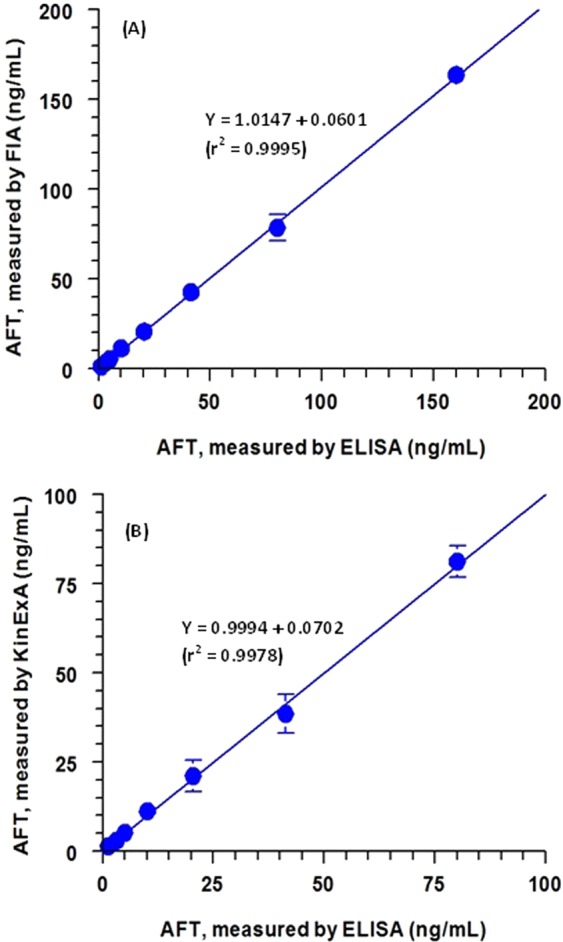
Correlations between AFT concentrations (in ng/mL) measured in plasma samples by ELISA with the same concentrations measured by FIA (**A**) and KinExA (**B**). Linear regression equations with determination coefficients (r^2^) are given on the fitting line of each particular correlation data.

To verify the reliability of the proposed FIA and KinExA assays, plasma samples were spiked with varying AFT concentrations (2–160 ng mL^−1^) and the samples were analyzed for AFT contents by a pre-validated reference method^16^ and by the proposed assays. Next, the results obtained by the three methods were subjected to regression statistical analysis (Fig. 9). We observed that the AFT concentrations measured by both FIA and KinExA were correlated very well with those measured by the reference method. In addition, the slopes of the curves correlating the results obtained by ELISA with those of FIA and KinExA are very close to 1 (1.0147 and 0.9994 in case of FIA and KinExA, respectively) and the intercepts of the graphs are close to zero (0.0601 and 0.0702 in case of FIA and KinExA, respectively). These results confirmed the reliability of the proposed FIA and KinExA for measurement of AFT concentrations in plasma samples.

The specificity of the anti-AFT antibody used in this study was confirmed in a previous study^16^; however, it is well known that immunoassay format may influence its cross reactivity. Therefore, the cross reactivity of drugs that might be used with AFT in targeted therapy for NSCLC was tested by the proposed competitive assays, FIA and KinExA. These drugs were crizotinib, certinib, alectinib, brigatinib, lorlatinib, erlotinib, gefitinib, dabrafenib and trametinib. We observed that none of these drugs cross react with the anti-AFT antibody in both FIA and KinExA under the respective optimized conditions.

### Comparative evaluation of FIA and KinExA with existing technologies

A summary of analytical performances for AFT bioanalysis between the proposed methods, FIA and KinExA with existing technologies is provided in Table 4. The data proved that KinExA had higher sensitivity (lower LOQ) than FIA; moreover, both assays had higher sensitivity than ELISA developed using the same reagents^16^. Because the three assays were developed by the same antibody and AFT-BSA conjugate, the higher sensitivity of both FIA and KinExA than that of ELISA was attributed to the high sensitivity of the fluorescence signals of FITC-IgG used in FIA and KinExA over the enzyme/chromogenic substrate used in ELISA. The higher sensitivity of KinExA over FIA and ELISA was attributed to the higher surface area of PMMA beads over that of the microwells in FIA and ELISA; the reported surface area of PMMA in KinExA is ~260 mm^2^, whereas the calculated surface area of each microwell in FIA is 64 mm^2^ ^29^. This high surface area maximizes the chances for capturing a high number of antibody molecules, ultimately enhancing the fluorescence signals. In addition, the rapid flow of anti-AFT antibody over the PMMA beads minimized the effective competition of immobilized AFT-BSA, thereby driving the antibody to bind to free AFT molecules in the samples and ultimately increasing the sensitivity of KinExA. Both FIA and KinExA had comparable accuracies of 101.8 ± 1.8 and 99.4 ± 3.4%, respectively. KinExA had higher precisions than FIA and ELISA with maximum RSD values of 8.4, 4.8 and 7.1%, respectively. The high precision of KinExA was due to the dependence of its precision on only the concentrations of anti-AFT and FITC-IgG antibodies which were dispended automatically with high precision by the KinExA instrument. In contrast, these reagents were manually dispensed in both FIA and ELISA. In addition, the differences in the uniformity of quantities of AFT-BSA immobilized onto the wells decreased the precisions of FIA and ELISA. This comparison confirmed that the format of the assay can influence its feastures even when the same reagents are used in its development.

**Table 4:** Comparison of FIA and KinExA with existing techniques for analysis of AFT in human plasma^a^.

Technique	Pretreatment of plasma samples	Range (ng mL^−1^)	LOQ (ng mL^−1^)	Accuracy (Recovery %)	Precision (RSD, %)	Reference
FIA	Dilution with PBS	0.008–200	1.2	101.8 ± 1.8	4.2–8.4	Present work
KinExA	Dilution with PBS	0.01–100	0.3	99.4 ± 3.4	4.2–4.8	Present work
ELISA	Dilution with PBS	0.04–2000	4	100.9 ± 1.42	3.5–7.1	^16^
UPLC-DAD	SPE	5–250	5	88.0 ± 2.2	1.63–5.65	^28^
LC-MS/MS	LLE	0.5–100	4.32	≥99.14	0.38–2.41	^10^
LC-MS/MS	LLE	0.05–500^b^	1.29	100.0 ± 2.8	1.53–4.11	^11^

^a^Abbreviations are:; FIA: fluoroimmunoassay, KinExA: kinetic exclusion assay, ELISA: enzyme-linked immunosorbent assay, UPLC-DAD: ultraperformace liquid chromatography with diode array detection, LC-MS/MS: liquid chromatography with tandem mass spectrometric detection, PBS: phosphate buffer saline, SPE: solid phase extraction, LLE: liquid-liquid extraction, LOQ: limit of quantitation, RSD: relative standard deviation, NA: not available.

^b^Values are in pg mL^−1^.

The immunoassays (FIA, KinExA and ELISA) had comparable accuracies with the existing chromatographic methods; however, their sensitivities were higher than those of most chromatographic methods, as indicated by the LOQ values. This enables immunoassays to quantify lower concentrations of AFT in pharmacokinetic studies particularly at the initial and terminal phases of pharmacokinetic profile. In addition, the pretreatment of plasma samples prior to analysis by immunoassays was just simple dilution with PBS; whereas, chromatographic methods required solid-phase or liquid-liquid extraction procedures. This simple pretreatment procedure of plasma samples enabled the high through capability and suitability of immunoassays for processing large number of samples in clinical settings.

## Conclusions

For the first time, two different formats of immunoassays, namely microwell plate-based FIA on fluorescence reader and KinExA on KinExA 3200 biosensor, were developed and validated for bioanalysis of AFT. Both assays showed different performance characteristics, despite being developed using the same reagents. Although, both assays had comparable sensitivities, KinExA had lower limit of quantitation and better coefficient of variation than FIA. As well, both FIA and KinExA assays were better than the available chromatographic assays for AFT as the proposed FIA and KinExA had higher sensitivity, simpler procedures, and they were more convenient. Both FIA and KinExA are expected to be useful in bioanalysis of AFT in clinical laboratories to increase the therapeutic benefits of AFT and decrease the potential side effects during therapy.

## References

[1] PubChem Afatinib dimaleate, https://pubchem.ncbi.nlm.nih.gov/compound/afatinibdimaleate#section=Top, Accessed 10 March 2019 (2017).

[2] Li D (2008). BIBW2992, an irreversible EGFR/HER2 inhibitor highly effective in preclinical lung cancer models. Oncogene.

[3] Mendelsohn J, Baselga J (2000). The EGF receptor family as targets for cancer therapy. Oncogene.

[4] Boehringer Ingelheim Pharmaceuticals, Inc. Gilotrif™ (afatinib) tablets for oral use. Initial U.S. Approval: 2013, www.accessdata.fda.gov/drugsatfdadocs/label/2013/201292s000lbl.pdf, Accessed 10 March 2019 (2017).

[5] Chemocare, Afatinib (gilotrif) chemotherapy drug information, http://chemocare.com/chemotherapy/drug-info/afatinib.aspx, Accessed 10 March 2019 (2017).

[6] Yu H (2014). Practical guidelines for therapeutic drug monitoring of anticancer tyrosine kinase inhibitors: focus on the pharmacokinetic targets. Clin. Pharmacokinet..

[7] de Jonge ME, Huitema AD, Schellens JH, Rodenhuis S, Beijnen JH (2005). Individualised cancer chemotherapy: strategies and performance of prospective studies on therapeutic drug monitoring with dose adaptation: a review. Clin. Pharmacokinet..

[8] US FDA, Center for Drug Evaluation and Research. Afatinib clinical pharmacology and biopharmaceutics review, www.accessdata.fda.gov/drugsatfdadocs/nda/2013/201292Orig1s000ClinPharmR.pdf. Accessed 10 March 2019 (2012).

[9] Picard S (2007). Trough imatinib plasma levels are associated with both cytogenetic and molecular responses to standard-dose imatinib in chronic myeloid leukemia. Blood.

[10] Abdelhameed AS, Attwa MW, Kadi AA (2017). An LC–MS/MS method for rapid and sensitive high-throughput simultaneous determination of various protein kinase inhibitors in human plasma. Biomed. Chromatogr..

[11] Kadi AA, Abdelhameed AS, Darwish HW, Attwa MW, Al-Shakliah NS (2016). A highly efficient and sensitive LC–MS/MS method for the determination of afatinib in human plasma: application to a metabolic stability study. Biomed. Chromatogr..

[12] Seger C (2012). Usage and limitations of liquid chromatography-tandem mass spectrometry (LC–MS/MS) in clinical routine laboratories. Wien, Med, Wochenschr.

[13] Yu Z, Tang Y, Cai G, Ren R, Tang D (2019). Paper electrode-based flexible pressure sensor for point-of-care immunoassay with digital multimeter. Anal. Chem..

[14] Luo Z (2019). Branched polyethylenimine-modified upconversion nanohybrid-mediated photoelectrochemical immunoassay with synergistic effect of dual-purpose copper ions. Anal. Chem..

[15] Lai W, Wei Q, Xu M, Zhuang J, Tang D (2017). Enzyme-controlled dissolution of MnO_2_ nanoflakes with enzyme cascade amplification for colorimetric immunoassay. Biosens. Bioelectron..

[16] Al-Shehri MM, El-Gendy MA, El-Azab AS, Hamidaddin MA, Darwish IA (2018). Development and validation of an ELISA with high sensitivity for therapeutic monitoring of afatinib. Bioanalysis.

[17] Kakabakos SE, Georgiou S, Petrou PS, Christofidis. (1999). Heterogeneous fluoroimmunoassays using fluorescein as label with measurement of the fluorescence signal directly onto the solid-phase. J. Immunol. Methods.

[18] Wood, P. Heterogeneous fluoroimmunoassay. In: Price, C. P., Newman, D. J. (Eds). Principles and practice of immunoassay, pp.365–392, 10.1007/978-1-349-11236-4, https://www.researchgate.net/publication/302448317_Heterogeneous_fluoroimmunoassay (1999)

[19] Hamidaddin MA, AlRabiah H, Darwish IA (2018). Development and validation of generic heterogeneous fluoroimmunoassay for bioanalysis of bevacizumab and cetuximab monoclonal antibodies used for cancer immunotherapy. Talanta.

[20] AlRabiaha H, Hamidaddin MA, Darwish IA (2019). Automated flow fluorescent noncompetitive immunoassay for measurement of human plasma levels of monoclonal antibodies used for immunotherapy of cancers with KinExA 3200 biosensor. Talanta.

[21] Darwish IA, Wani TA, Hamidaddin MA (2018). Development of highly Efficient KinExA immunosensor-based assay for the measurement of carcinoembryonic antigen in serum. Curr. Anal. Chem..

[22] Wani TA, Alanazi AM, Hamidaddin MA, Zargar S, Darwish IA (2013). Kinetic-exclusion analysis-based immunosensors versus enzyme-linked immunosorbent assays for measurement of cancer markers in biological specimens. Talanta.

[23] Mastronicolis SK, Kapoulas VM, Kröger H (1981). The isolation of antibodies specific for 5-methyl-cytidine-bovine serum albumin. Z. Naturforsch.

[24] Darwish IA, Al-Obaid AM, Al-Malaq HA (2011). Generation of polyclonal antibody with high avidity to rosuvastatin and its use in development of highly sensitive ELISA for determination of rosuvastatin in plasma. Chem. Cent, J..

[25] Alzoman NZ, Darwish IA, Abuhejail RM (2014). A highly sensitive polyclonal antibody-based ELISA for therapeutic monitoring and pharmacokinetic studies of lenalidomide. J. Immunoassay Immunochem..

[26] Findlay JW (2000). Validation of immunoassays for bioanalysis: a pharmaceutical industry perspective. J. Pharm. Biomed. Anal..

[27] Aanderson DJ (1989). Determination of the lower limit of detection. Clin. Chem..

[28] Fouad M, Helvenstein M, Blankert B (2015). Ultra-high performance liquid chromatography method for the determination of two recently FDA approved TKIs in human plasma using diode array detection. J. Anal. Methods Chem..

[29] Blake RC, Pavlov AR, Blake DA (1999). Automated kinetic exclusion assays to quantify protein binding interactions in homogeneous solution. Anal. Biochem..

